# Citrus Irrigation With Desalinated Seawater Under a Climate Change Scenario

**DOI:** 10.3389/fpls.2022.909083

**Published:** 2022-05-30

**Authors:** Josefa María Navarro, Vera Antolinos, Juan Miguel Robles, Pablo Botía

**Affiliations:** Irrigation and Stress Physiology Group, Department of Bioeconomy, Water and Environment, Murcia Institute of Agri-Food Research and Development (IMIDA), Murcia, Spain

**Keywords:** *Citrus macrophylla*, sour orange, lemon, boron, sodium, chloride

## Abstract

In arid and semiarid regions, the current lack of natural water resources is driving the use of alternative sources for crop irrigation, such as desalinated seawater (DSW). However, the use of DSW could affect the crop productivity due to its chemical composition (predominance of phytotoxic ions: Na^+^, Cl^−^, and B). Citrus species are classified as salt and boron-sensitive; however, the rootstock plays a fundamental role in the tree’s tolerance of abiotic stresses. One-year-old ‘Verna’ lemon trees grafted on two rootstocks (CM, *Citrus macrophylla*, and SO, sour orange) were used. These rootstocks differ in their salinity and boron tolerance, SO being more tolerant than CM. The experiment was carried out at high temperature (35/27°C), and the plants were irrigated with three types of water supplemented with Hoagland nutrients: DSW, DLB (DSW with low boron), and Control (distilled water). The plants were irrigated three times per week and harvested 7 months after the treatments started. The response to high levels of Cl^−^, Na^+^, and B was rootstock-dependent. Under the high temperature conditions, the growth of plants grafted on SO was not affected by DSW, and these plants did not reach the Cl^−^ threshold of phytotoxicity, so the decrease in the shoot growth of plants grafted on CM due to DSW irrigation was related more to Cl^−^ rather than the foliar Na^+^ accumulation. Plants grafted on SO and irrigated with DSW accumulated more B than those grafted on CM, surpassing the threshold of phytotoxicity and producing greater oxidative stress. As the growth of these plants was not reduced, the effects of DSW on plant growth were not directly related to the concentration of B and there must be some mechanisms that allow these plants to withstand the negative effects of high foliar B, such as the increased levels of quaternary ammonium compounds. Since the response of citrus plants to DSW depended on the rootstock, the results obtained in this experiment, using DSW at high temperature, could be useful for the future management of citrus crops, because climate change will increase temperatures and exacerbate the scarcity of water resources in citrus-growing areas.

## Introduction

Citrus is one of the most important horticultural crops in the world, widely cultivated in Southeastern Spain where the climate conditions are those typical of the semiarid zone, with low rainfall, dry and hot summers, and high evaporative demand. Moreover, the high structural deficit of water suffered in this area (about 429 hm^3^ per year, CHS, 2015) makes it the one with the highest water deficit in the EU. Furthermore, as a result of climate change (CC), it is expected that by mid-century the availability of water will have declined by 40% in some regions. Hence, the use of non-conventional water resources is needed, and the Intergovernmental Panel on Climate Change (IPCC) has proposed desalination of seawater as a potential option to adapt agriculture to the impacts of CC, especially in arid and semiarid regions (Bates et al., 2008).

However, the composition of desalinated seawater (DSW) differs markedly from those of conventional water sources used for irrigation in Southeastern Spain (Martínez-Alvarez et al., 2017) and the agronomic effects of its incorporation into crop irrigation depend on the quality of the irrigation water that is replaced. Special attention should be paid when DSW replaces high-quality waters since, contrary to what happens when low-quality waters are replaced, its electrical conductivity (EC) does not represent an improvement, and there are agronomic risks due to its characteristic chemical composition, quite different from those of conventional water sources (Martínez-Alvarez et al., 2016). DSW produced through reverse osmosis has low mineralization since some nutrients essential for plants that are generally abundant in natural waters (Mg^2+^, Ca^2+^, and SO_4_^2−^) have been partially removed. Moreover, the predominant ions that remain in DSW are sodium (Na^+^) and chloride (Cl^−^), while boron (B) also has a high presence (Martínez-Alvarez et al., 2016, 2017).

Citrus plants are sensitive to the toxic effects of Cl^−^ and/or Na^+^ accumulation in the leaves and relatively low salinity levels lead to physiological disturbance and a reduction in growth and fruit yield (Maas, 1993; Storey and Walker, 1999). High concentrations of Cl^−^ and Na^+^ in leaves can damage plants by reducing the net assimilation of CO_2_, due to a direct biochemical inhibition of the photosynthetic process (Garcia-Sanchez and Syvertsen, 2006), while nutritional imbalances can arise under salinity due to the lower uptake of some nutrients (Grattan and Grieve, 1992; Romero-Aranda et al., 1998). In addition, the high B concentration in DSW is worrying in the case of citrus crops since they are very sensitive to excess B. In general, waters with a B concentration above 0.5 mg L^−1^ can create toxicity problems (Voutchkov and Semiat, 2008; Martínez-Alvarez et al., 2017). Under B toxicity conditions, trees show reduced vigor, delayed development, and reduced fruit number and weight, as well as chlorotic and necrotic spots in older leaves and premature leaf abscission (Nable et al., 1997). To mitigate the risk of crop damage due to B in some special cases, such as sensitive and valuable crops, its concentration in DSW could be reduced prior to the use of DSW for irrigation. On-farm systems designed to reduce the B concentration of DSW by the use of B-selective ion exchange resins or ultrafiltration membranes have been studied (Darwish et al., 2015; Imbernón-Mulero et al., 2022). Although the interaction of Cl^−^, Na^+^, and B has been studied in several plant species, inconsistent results in relation to B supply levels and concomitant differences in predominant B uptake pathways have been found (Wimmer and Goldbach, 2012). In citrus, a decrease in leaf B accumulation was found when seedlings were supplied with saline water (Cooper et al., 1952). In citrus trees irrigated with recycled wastewaters (high EC and B concentrations), the leaf B concentration increased in a short period of time (Pedrero and Alarcon, 2009; Pedrero et al., 2012). In recent studies, in which DSW was used to irrigate citrus trees, no differences were found in the foliar accumulation of phytotoxic elements, physiological responses, or fruit yield, probably due to the short-term nature of the experiment (Maestre-Valero et al., 2020). Unfortunately, there is not much information available regarding the combined effects of high concentrations of Cl^−^, Na^+^, and B in citrus plants.

Under adverse environmental conditions, one of the agronomic strategies employed to increment the stress tolerance of citrus is the use of tolerant rootstocks (Syvertsen and Garcia-Sanchez, 2014). The salinity tolerance of citrus trees is related to the ability of citrus rootstocks to restrict the uptake of Cl^−^ and Na^+^ and/or their transport from roots to shoots (Maas, 1993; Romero-Aranda et al., 1998; García-Sánchez et al., 2002). Common commercial citrus rootstocks can be classified based on their degree of accumulation/exclusion of Cl^−^ and Na^+^: Citrus macrophylla (*Citrus macrophylla* Wester) is considered to be an accumulator of both Cl^−^ and Na^+^, Carrizo citrange (*Citrus sinensis* Osb. × *Poncirus trifoliate* L. Raf.) is a Na^+^ excluder, Cleopatra mandarin (*Citrus reticulata* Blanco) is a good Cl^−^ excluder, and sour orange (*Citrus aurantium* L.) is considered a good Cl^−^ and Na^+^ excluder (Nieves et al., 1991; Storey and Walker, 1999). The rootstock also plays a fundamental role in the citrus tolerance of B since it can restrict the uptake of B and/or its transport from the roots to the leaves, limiting its accumulation in the leaves and minimizing its toxic effect (Papadakis et al., 2004; Gimeno et al., 2012; Mesquita et al., 2016).

The simultaneous occurrence of several stresses can cause a new stress situation in which the effects on plants are more negative than for individual stresses (Zandalinas et al., 2018; Balfagón et al., 2021). In this sense, some studies have shown that the combination of high temperatures with salinity or drought can be lethal to citrus plants (Zandalinas et al., 2018; Balfagón et al., 2021). The increases in the temperatures throughout this century anticipated by the IPCC for Southeastern Spain will suppose an important danger for citriculture in this area. To our knowledge, there are no data available regarding the effect of irrigation with DSW on citrus plant behavior under high temperature conditions. Since DSW has been adopted as an alternative source of water in areas with serious water scarcity, where citrus production is essential for economic and social sustainability, and because CC is expected to aggravate this situation, more knowledge about the effects of the use of DSW for irrigation in citrus cultivation is necessary. The aim of the present research was to study the effects of irrigation with DSW on the accumulation of phytotoxic elements, plant growth, mineral nutrition, and physiology of citrus (lemon) plants grown at high temperature under controlled conditions. In an attempt to better understand the behavior of rootstocks that show different responses to salinity and boron, we studied the rootstocks used most commonly in lemon orchards in Southeastern Spain, namely sour orange (*Citrus aurantium*) and *Citrus macrophylla*, which show different levels of tolerance of Cl^−^, Na^+^, and B. We also evaluated the effect of irrigation with DSW, with a reduced boron concentration, produced by a reverse osmosis system. The differences between the SO and CM rootstocks in their Cl^−^, Na^+^, and B tolerance could modify the behavior of SO and CM-grafted plants (involving nutritional, physiological, and biochemical alterations) when they are irrigated with desalinated seawater (DSW) and desalinated seawater with a low B concentration (DLB).

## Materials and Methods

### Plant Material, Growing Conditions, and Experimental Design

One-year-old ‘Verna’ lemon (*Citrus limon* Burm. f. cv. Verna) trees grafted on two different rootstocks, *Citrus macrophylla* (CM) and sour orange (SO), were used in this experiment. The plants, acquired from a commercial nursery, were transplanted to 3-L-capacity pots filled with a substrate composed of a mixture of silica filtration sand (particle size 0.4–0.8 mm) and clay-loam soil (soil:sand 3:1, v/v). The experiment was carried out in a walk-in controlled environment room (3 m × 6.5 m) at the IMIDA, where we tried to simulate the field conditions in Southeastern Spain predicted by the IPCC (IPCC 2019)^1^ in its most extreme scenario of CC, with an increase of nearly 6°C in the average temperature during summer in this area. For that, the meteorological data recorded during the 2015–2019 period by a meteorological station in Murcia (37.747654, −0.986830) were used to calculate the current mean day/night temperatures, relative humidity (RH), and photoperiod duration. To simulate the temperature increase due to CC, 6°C were added to these mean day/night temperatures. The experiment was carried out under a 14/10 h day/night cycle, with temperatures of 35/27°C and RH of 55/85%. After the plants had acclimatized to these conditions for 2 weeks, the irrigation treatments were applied.

The experimental treatments consisted of factorial combinations of two factors: the first one was the nutrient solution that consisted of three types of water, supplemented with Hoagland nutrients (Hoagland and Arnon, 1950)—distilled water (Control), desalinated seawater (DSW), and DSW with low B (DLB)—and the second was the rootstocks, CM and SO. Boron was not added to the DSW or DLB. The final mineral concentrations in the three types of nutrient solutions are shown in Table 1. The DSW was obtained from the desalination plant of Escombreras (Murcia, Spain). The DLB, obtained by passing DSW through a reverse osmosis system, had a reduced B concentration. Each of the six treatments had four replicates, giving a total of 24 pots. The experiment was laid out in a completely randomized design and the positions of the pots were changed every week to eliminate environmental variation.

**Table 1:** Average ionic composition and electrical conductivity (EC) of the nutrient solutions in the Control, DSW (desalinated seawater), and DLB (desalinated seawater with low boron) treatments used for irrigation during the experiment.

	Control	DSW	DLB
EC (μS cm^−1^)	2,000	3,079	2,067
Cl^−^ (mg L^−1^)	1.8	300.1	10.2
Na^+^ (mg L^−1^)	0.0	166.6	6.9
B (mg L^−1^)	0.27	1.23	0.50
NO_3_^−^ (mg L^−1^)	992	1,003	998
H_2_PO_4_^−^ (mg L^−1^)	192	192	192
SO_4_^2−^ (mg L^−1^)	96.0	108.3	103.2
K^+^ (mg L^−1^)	235.0	240.3	235.2
Ca^2+^ (mg L^−1^)	160.0	179.3	162.5
Mg^2+^ (mg L^−1^)	24.0	28.6	26.1
Cu (mg L^−1^)	0.032	0.044	0.043
Zn (mg L^−1^)	0.131	0.131	0.131
Mn (mg L^−1^)	0.11	0.11	0.11
Fe (mg L^−1^)	1.12	1.12	1.12

The plants were irrigated three times per week, with 400 ml/pot (a volume sufficient to produce leachate from the bottom of all pots) of the DSW, DLB, and Control solutions, and were harvested 7 months after the treatments started.

### Physiological Parameters Determination

Gas exchange parameters, chlorophyll fluorescence, and leaf water relations were measured fortnightly. Leaf gas exchange was measured in the youngest fully expanded leaf of each plant, using a portable photosynthesis system (Li-6,400, Li-Cor, Lincoln, Nebraska, United States) equipped with a broad leaf chamber (6.0 cm^2^). The air flow rate inside the leaf chamber was 300 μmol s^−1^. Portable 12-g cartridges of high-pressure, liquefied, pure CO_2_ were attached to the console through an external CO_2_ source assembly and they were controlled automatically by a CO_2_ injector system, which fixed the CO_2_ concentration at 400 μmol CO_2_ mol^−1^. All the measurements were made using a red-blue light source attached to the leaf chamber and the PPFD was fixed at 1000 μmol m^−2^ s^−1^.

Chlorophyll fluorescence measurements were performed in parallel to those of the gas exchange parameters, on the same days and in the same leaves, using a pulse-modulated field-fluorescence monitoring system (FMS-2, Hansatech Instruments, Norfolk, United Kingdom). Leaves were previously adapted to darkness for 30 min and subsequently illuminated for 5 μs to calculate the ratio (*F*_m_ − F_0_)/*F*_m_, where *F*_0_ and *F*_m_ are the initial and the maximum fluorescence, respectively. The chlorophyll fluorescence kinetics of leaves adapted to light were also studied, and the following parameters were measured: the quantum efficiency of PSII [*Φ*_PSII_ = (*F*_m_′ − *F*_s_)/*F*_m_′], the antennae efficiency of PSII [*F*_v_′/*F*_m_′ = (*F*_m_′ − *F*_0_′)/*F*_m_′], and the photochemical quenching coefficient [qP = (*F*_m_′ − *F*_s_)/(*F*_m_′ − *F*_0_′)], where *F*_s_ is the steady-state fluorescence yield, *F*_m_′ is the maximal value when all reaction centers are closed after a pulse of saturating light (12,000 μmol m^−2^ s^−1^ for 0.8 s), and *F*_0_′ is the minimal fluorescence in the light-adapted state that is obtained by turning off the actinic light temporarily and applying a pulse of far-red light (735 nm) to drain the electrons from PSII.

The leaf water potential (Ψ_leaf_) was measured in mature fully expanded leaves with a Schölander-type pressure chamber (model 3000; Soil Moisture Equipment Corp., Santa Bárbara, California, United States), following the recommendations of Turner (1988). Following Ψ_leaf_ measurement, the leaves were immediately frozen and stored at −20°C for determination of the leaf osmotic potential (Ψ_Π_) by a Wescor 5520 vapor pressure osmometer (Wescor, Logan, UT, United States). The leaf turgor potential was calculated as the difference between Ψ_leaf_ and Ψ_Π_.

### Plant Growth and Mineral Analysis

At the end of the experiment, the roots were carefully separated from the substrate and washed with distilled water. The shoots were separated into leaves and stems, which were also divided into lateral (growth after transplanting) and old stems. The leaf area of each plant was measured using a leaf area meter (model LI-3100, Li-Cor, Lincoln, NE, United States). Each plant material fraction was weighed fresh (FW) and after oven-drying for 48 h to determine the dry weight (DW). A sample of leaves was freeze-dried for metabolites determination.

The dried plant tissues were ground and an aliquot (250 mg) was ashed at 550°C. The ashes were dissolved in 0.7 N HNO_3_, and macronutrients (P, K, Ca, and Mg), micronutrients (Fe, Cu, Mn, and Zn), and phytotoxic elements (Na and B) were determined by inductively coupled plasma optical emission spectrometry (Varian ICP-OES Vista MPX). Chloride and NO_3_^−^ were extracted from 50 mg of ground plant material with 25 ml of deionized water and measured by ion chromatography with a liquid chromatograph (Model ICS-3000, Thermo Fisher Scientific Inc., United States). The nitrogen concentration was determined with a LECO FP-428 protein detector.

### Leaf Metabolites Determination

At the end of the experiment healthy, fully expanded mature leaves were sampled, then freeze-dried and ground for analytical determinations. Proline was extracted from 50 mg of leaf tissue with sulfosalicylic acid (3%) and quantified according to the protocol described by Bates et al. (1973). Quaternary ammonium compounds (QACs) were extracted from dry tissue with 1 M H_2_SO_4_ and were quantified using a glycine-betaine standard curve, according to the method described in Grieve and Grattan (1983).

The chlorophyll contents were estimated using the procedure described by Inskeep and Bloom (1985), extracting 20 mg of ground material with N,N-dimethylformamide and measuring the absorbance at 664.5 and 647 nm in a Shimadzu UV-1800 spectrophotometer (Shimadzu Corporation, Kioto Japan).

### Leaf Injury

Before harvesting the plants, the leaf H_2_O_2_ was determined by following the method described by Velikova et al. (2000), using trichloroacetic acid (TCA) as the extracting agent. To this end, 250 mg of fresh leaf tissue were homogenized in 0.1% TCA. After centrifugation at 12,000 rpm for 15 min at 4°C, 0.5 ml of the filtered supernatant (0.45 μm) was added to 0.5 ml of 10 mM potassium phosphate buffer (pH 7) and 1 ml of 1 M KI. The absorbance of this mixture was read at 390 nm.

Lipid peroxidation was determined by measuring malondialdehyde (MDA), using the Heath and Packer (1968) method as modified by Du and Bramlage (1992). Dry tissue was homogenized with a thiobarbituric acid–TCA mixture, and the absorbance was read at 532 nm, 600 nm (for correction of nonspecific turbidity), and 440 nm (to rectify the interference of soluble sugars in the samples).

### Substrate Analysis

Chemical characterization of the substrate was performed at the beginning and end of the experiment, in accordance with Bower and Wilcox (1965). The following parameters were analyzed in a substrate/water (1/5) extract: EC, pH, exchangeable cations (Ca^2+^, Mg^2+^, K^+^, and Na^+^), P, B, micronutrients (Fe, Mn, Cu, and Zn), and anions (Cl^−^, SO_4_^2−^, NO_3_^−^, and H_2_PO_4_^−^).

### Statistical Analysis

The data were analyzed using analysis of variance (ANOVA) procedures in Statgraphics Plus 5.1 software (Statistical Graphics Corporation, Warrenton, VA, United States). A two-way ANOVA procedure was used to discriminate the effects of the rootstock and irrigation method. When there was a significant effect (value of *p* < 0.05), means were separated using Duncan’s multiple range test.

## Results

### Impact of Treatments on Substrate Chemical Properties

After 7 months of irrigation with the Control, DSW, or DLB treatment, the EC was higher in the substrates of all three treatments than in the substrate before the experiment began (Table 2). Besides, at the end of the experiment, DSW-irrigated substrates had significantly higher EC than those irrigated with Control or DLB water. This increase was due to the higher levels of Na^+^, Cl^−^, and B in the soil solution (Table 2). Besides, the B concentrations in substrates of CM plants were significantly higher than those in substrates of SO plants (data not shown).

**Table 2:** Substrate chemical properties for the initial substrate (before the experiment) and at the end of the experiment, after 7 months of irrigation with nutrient solutions of different characteristics: Control, DSW, and DLB.

	Before experiment	End of experiment			ANOVA (end of experiment)		
		Control	DSW	DLB	Nutrient solutions (NS)	Rootstock (R)	NS × R
EC (μS cm^−1^)	206	271a	349b	286a	*	ns	ns
pH	8.6	8.4	8.3	8.2	ns	ns	ns
Na (mg kg^−1^)	90.3	505b	91.8c	17.4a	***	ns	ns
Cl^−^ (mg kg^−1^)	97.9	44.0a	107.1b	13.6a	***	ns	ns
B (mg kg^−1^)	0.30	0.29a	0.98b	0.30a	***	**	ns
Ca (mg kg^−1^)	115	125	131	128	ns	ns	ns
Mg (mg kg^−1^)	16.5	18.3	18.2	17.5	ns	ns	ns
K (mg kg^−1^)	23.0	130	144	138	ns	ns	ns
P (mg kg^−1^)	2.19	5.04	4.10	3.11	ns	ns	ns
Fe (mg kg^−1^)	1.59	2.00b	1.25ab	1.16a	*	ns	ns
Cu (μg kg^−1^)	75.0	67.4	66.3	56.6	ns	ns	ns
Zn (μg kg^−1^)	0.50	5.34	1.26	0.24	ns	ns	ns
Mn (μg kg^−1^)	827.2	47.0	42.4	12.8	ns	ns	ns
NO_3_^−^ (mg kg^−1^)	17.1	188a	289b	342b	*	ns	ns
H_2_PO_4_^−^ (mg kg^−1^)	0.15	7.23	6.15	2.99	ns	ns	ns
SO_4_^2−^ (mg kg^−1^)	57.1	108	123	119	ns	ns	ns

^*^p < 0.05; ^**^p < 0.01; ^***^p < 0.001. ns, not significant. Different letters indicate significant differences according to Duncan’s multiple range test at the 95% CI.

### Plant Growth

Under the high temperature conditions of this experiment, the use of DSW decreased the shoot growth of the citrus plants with respect to Control or DLB-irrigated plants (Table 3). This lower shoot growth and the slight increase in root growth produced an increase in the root/shoot ratio in DSW-irrigated plants relative to Control plants. Besides, the shoot growth reduction due to irrigation with DSW was rootstock dependent, being greater in CM-grafted plants (19% and 35% for the dry weight of leaves and lateral stems, respectively) than in SO-grafted plants (3% and 18%, respectively). The growth reduction of shoots caused by DSW-irrigation was due to both lower stem weight and lower total leaf area (mainly due to the reduction of leaf size). Otherwise, in general, the irrigation for 7 months with DLB did not modify the biometric parameters studied, except the leaf size and the root/shoot ratio (Table 3).

**Table 3:** Effects of 7 months of irrigation with nutrient solutions of different characteristics—Control, DSW, and DLB—and the rootstock on different parameters of the growth, and on the root/shoot weight ratio, of ‘Verna’ lemon plants grown on two rootstocks, *Citrus macrophylla* (CM) and sour orange (SO).

Nutrient solution (NS)		Dry weight (g plant^−1^)					Root/shoot	Root/leaf	Lateral stems length (cm)	Mean leaf area (cm^2^ leaf^−1^)	Total leaf area (cm^2^ plant^−1^)	Leaf number
		Leaves	Lateral stems	Shoot	Roots	Total plants						
Control		39.2	16.4b	80.7b	16.0	95.6	0.225a	0.46	199	33.3b	4,300	136
DSW		34.5	12.3a	67.4a	18.7	86.1	0.278b	0.54	162	29.5a	3,781	134
DLB		38.5	15.6b	78.6b	18.0	94.3	0.260b	0.51	198	28.8a	4,354	148
Rootstock (R)												
*Citrus macrophylla* (CM)		40.1	14.6	76.2	16.4	90.2	0.223	0.41	210	32.7	4,692	151
Sour orange (SO)		34.7	15.0	75.0	18.8	93.9	0.285	0.60	163	28.4	3,598	127
R × NS												
CM	Control	42.8	16.6	79.3bc	15.0	94.3	0.188	0.35	195	36.7	4,951	146
	DSW	34.5	10.8	63.9a	15.9	79.7	0.249	0.46	200	30.7	4,041	143
	DLB	43.1	16.4	85.3c	18.2	96.5	0.232	0.42	233	30.6	5,084	165
SO	Control	35.6	16.3	82.2bc	17.0	97.0	0.262	0.57	203	30.0	3,649	126
	DSW	34.5	13.9	70.9ab	21.5	92.4	0.307	0.63	124	28.3	3,521	125
	DLB	33.9	14.7	71.9ab	17.8	92.2	0.287	0.60	163	27.0	3,624	131
ANOVA												
Nutrient solution (NS)		ns	*	**	ns	ns	**	ns	ns	*	ns	ns
Rootstock (R)		*	ns	ns	ns	ns	**	***	*	**	**	*
R × NS		ns	ns	*	ns	ns	ns	ns	ns	ns	ns	ns

^*^p < 0.05; ^**^p < 0.01; ^***^p < 0.001. ns, not significant. Different letters indicate significant differences according to Duncan’s multiple range test at the 95% CI.

Although the experiment commenced with plants of uniform size on both rootstocks, after 7 months, and regardless of the treatment, ‘Verna’ lemon plants on SO rootstocks had shorter stems (new growth), fewer leaves, and a lower leaf size that produced a lower total leaf area and leaf weight than in plants grafted on CM rootstocks (Table 3).

### The Accumulation and Partitioning of Phytotoxic Elements

**Figure 1:**
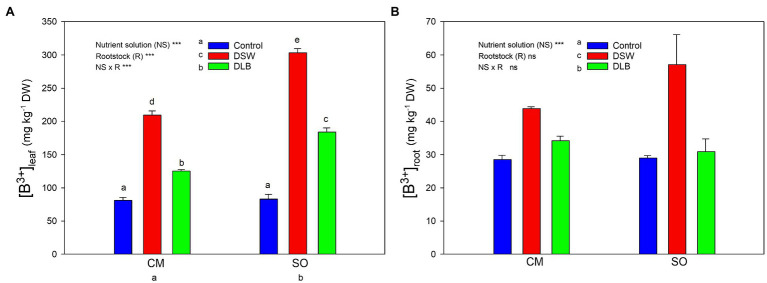
Effects of 7 months of irrigation with nutrient solutions of different characteristics—Control, desalinated seawater (DSW), and DSW with low boron (DLB)—on the B concentration in leaves **(A)** and roots **(B)** of ‘Verna’ lemon plants grown on two rootstocks, *Citrus macrophylla* (CM) and sour orange (SO). Different letters indicate significant differences according to Duncan’s multiple range test at the 95% confidence level.

**Figure 2:**
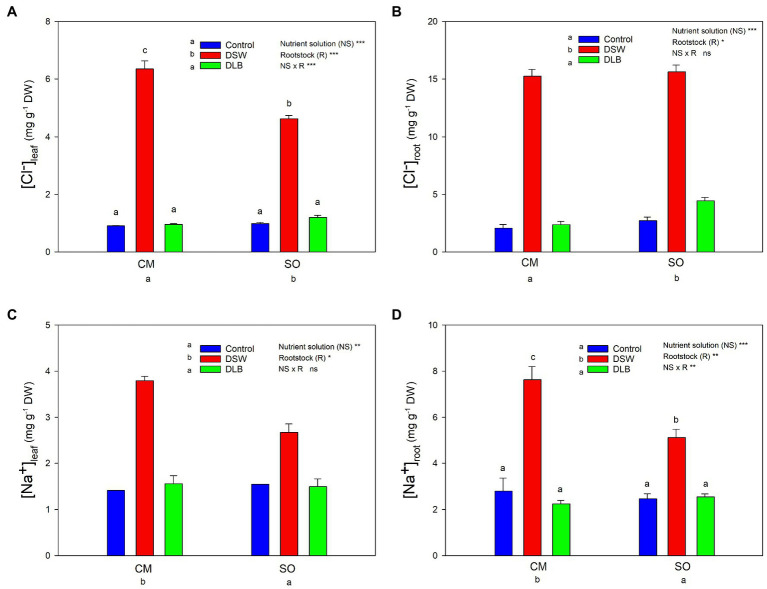
Effects of 7 months of irrigation with nutrient solutions of different characteristics—Control, DSW, and DLB—on the Cl^−^ **(A,B)** and Na^+^ **(C,D)** concentrations in leaves and roots of ‘Verna’ lemon plants grown on two rootstocks, *Citrus macrophylla* (CM) and sour orange (SO). Different letters indicate significant differences according to Duncan’s multiple range test at the 95% confidence level.

The accumulation of B by the ‘Verna’ lemon plants depended on the irrigation treatment, the rootstock, and the organ of the plant studied (Figure 1). In general, roots had significantly lower B concentrations than leaves, which accumulated approximately five times more B than roots. With regard to the irrigation treatments, DSW and DLB-irrigated plants accumulated more B in their leaves than Control plants, DSW being the treatment that gave the highest B values. Besides, the behaviors of the rootstocks differed; in comparison with CM, SO led to greater B accumulation in photosynthetically active leaves but not in roots (Figure 2).

After 7 months, the Cl^−^ and Na^+^ accumulation in leaves and roots of ‘Verna’ lemon plants varied according to the rootstock and irrigation treatment (Figure 2). In general, Cl^−^ accumulation in leaves and roots was higher than that of Na^+^ in these organs, the roots being the organ that accumulated more Cl^−^ and Na^+^ (Figure 2). Plants irrigated with DSW had higher leaf and root concentrations of Cl^−^ and Na^+^ than Control plants, whereas no differences were found between Control and DLB plants in the concentrations of Cl^−^ or Na^+^ in photosynthetically active leaves or roots. The rootstock significantly influenced the Cl^−^ and Na^+^ accumulation in the tissues: CM plants accumulated more Na^+^ (roots and leaves) and more Cl^−^ (leaves) than SO plants (Figure 2). The Cl^−^ concentrations in leaves of CM and SO plants irrigated with DSW were, respectively, eight and five times the concentrations of their respective Controls, whereas in roots they were seven and six times higher for CM and SO plants, respectively. Plants irrigated with DSW accumulated three (CM) and two (SO) times more Na^+^ in both leaves and roots than the respective Control plants (Figure 2).

**Figure 3:**
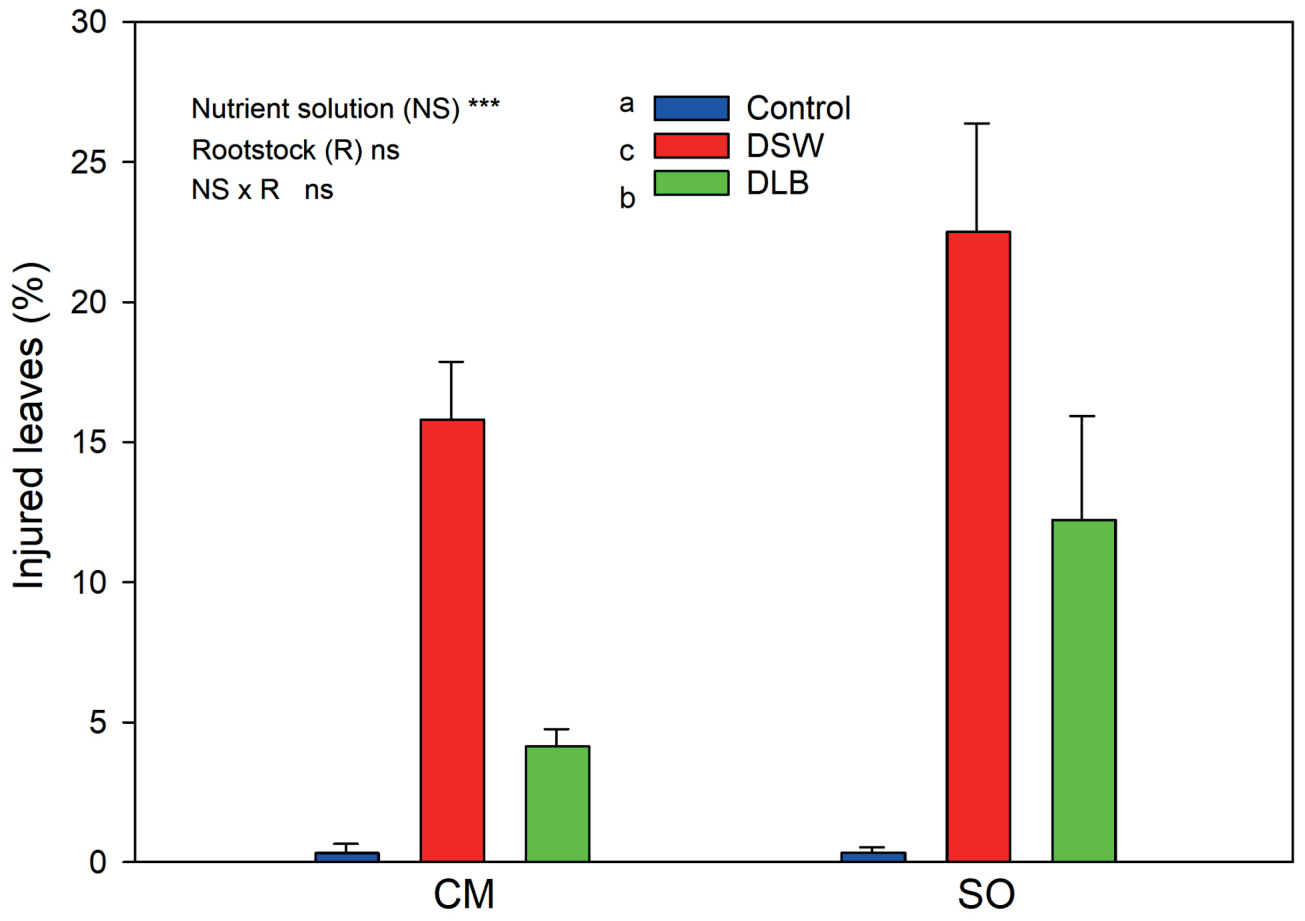
Effects of 7 months of irrigation with nutrient solutions of different characteristics—Control, DSW, and DLB—on the percentage of injured leaves of ‘Verna’ lemon plants grown on two rootstocks, *Citrus macrophylla* (CM) and sour orange (SO).

**Figure 4:**
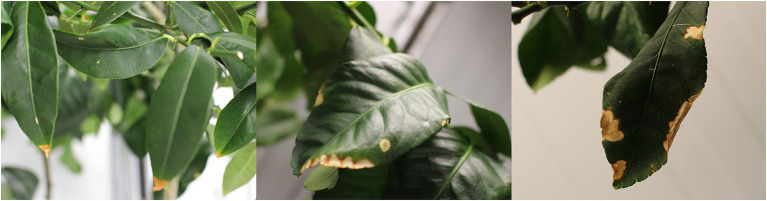
Visual damage to the leaves after 7 months of irrigation with nutrient solutions of different characteristics—Control, DSW, and DLB—for ‘Verna’ lemon plants grown on two rootstocks, *Citrus macrophylla* (CM) and sour orange (SO).

At the end of the experiment, visual injuries of leaves were observed, mainly in DSW-irrigated plants (19% of their leaves were affected) but also in DLB-irrigated plants (8%; Figure 3). These injuries were more or less severe depending on the treatment and the leaf age, but mainly consisted of necrosis along the tips and margins of leaves (Figure 4). Sour orange plants irrigated with DSW seemed to have more injuries, but the differences in comparison with CM plants were not significant.

### Plant Mineral Concentrations and Partitioning

At the end of the experiment, some differences in mineral nutrition were found in leaves and roots as a consequence of the irrigation with nutrient solutions of different mineral composition (Tables 4 and 5). However, there were also significant differences in the mineral status of the ‘Verna’ lemon trees due to the rootstocks, mainly in the roots. In general, the mineral concentrations in ‘Verna’ lemon plants grafted on SO were lower than in plants grafted on CM. For example, plants grafted on SO had lower levels of N, K, Fe, Cu, and Mn in their roots (Table 5) and lower N, K, Fe, Mn, and Zn in their leaves (Table 4). However, P, Ca, Mg, or Zn were present at higher concentrations in roots of SO than in roots of CM.

**Table 4:** Effects of 7 months of irrigation with nutrient solutions of different characteristics—Control, DSW, and DLB—and the rootstock on the leaf mineral nutrition of ‘Verna’ lemon plants grown on two rootstocks, *Citrus macrophylla* (CM) and sour orange (SO).

Nutrient solution (NS)		N	P	K	Ca	Mg	Fe	Cu	Mn	Zn
Control		3.8b	0.087	4.5	2.10b	0.21	106a	19.1a	39.0a	37.4
DSW		3.5a	0.094	4.5	1.83a	0.23	137b	30.0b	53.0b	41.5
DLB		3.7b	0.091	4.4	2.20b	0.23	111a	18.7a	41.5a	40.4
Rootstock (R)										
*Citrus macrophylla* (CM)		4.0	0.087	4.6	2.11	0.21	133	24.2	59.4	43.4
Sour orange (SO)		3.4	0.094	4.3	1.97	0.24	104	20.9	29.6	36.2
R × NS										
CM	Control	4.0	0.084	4.4ab	2.16	0.22	116	20.7	52.3	37.1
	DSW	3.8	0.082	4.6b	1.96	0.22	161	32.3	72.3	45.3
	DLB	4.1	0.095	4.8b	2.22	0.21	121	19.7	53.5	47.6
SO	Control	3.5	0.089	4.6b	2.03	0.21	96	17.4	25.6	37.7
	DSW	3.3	0.106	4.3ab	1.70	0.24	113	27.7	33.7	37.6
	DLB	3.3	0.087	4.0a	2.19	0.25	102	17.6	29.6	33.2
ANOVA										
Nutrient solution (NS)		*	ns	ns	**	ns	*	**	*	ns
Rootstock (R)		***	ns	*	ns	ns	**	ns	***	**
R × NS		ns	ns	*	ns	ns	ns	ns	ns	ns

N, P, K, Ca, Mg, and Na in g 100 g^−1^ DW; Fe, Cu, Mn, Zn, and B in mg kg^−1^DW. ^*^p < 0.05; ^**^p < 0.01; ^***^p < 0.001. ns, not significant. Different letters indicate significant differences according to Duncan’s multiple range test at the 95% CI.

**Table 5:** Effects of 7 months of irrigation with nutrient solutions of different characteristics—Control, DSW, and DLB—and the rootstock on the root mineral nutrition of ‘Verna’ lemon plants grown on two rootstocks, *Citrus macrophylla* (CM) and sour orange (SO).

Nutrient solution (NS)		N	P	K	Ca	Mg	Fe	Cu	Mn	Zn
Control		3.1b	0.442	2.1	2.9	0.26	1606b	69.2	292	55.3
DSW		2.7a	0.452	1.9	2.7	0.22	1169a	81.8	349	74.1
DLB		3.1b	0.392	2.1	2.5	0.23	1321a	66.4	289	60.4
Rootstock (R)										
*Citrus macrophylla* (CM)		3.3	0.282	2.3	2.1	0.22	1,464	89.4	475	51.9
Sour orange (SO)		2.6	0.575	1.8	3.3	0.25	1,267	55.5	146	74.6
R × NS										
CM	Control	3.5	0.261	2.2	2.2	0.24	1,609	80.7ab	438	49.6
	DSW	3.1	0.347	2.3	2.1	0.21	1,273	110.7c	545	59.8
	DLB	3.3	0.239	2.4	2.1	0.21	1,509	76.9b	443	46.4
SO	Control	2.6	0.623	1.9	3.7	0.27	1,602	57.8ab	147	61.0
	DSW	2.3	0.557	1.6	3.4	0.23	1,064	52.9a	154	88.4
	DLB	2.9	0.545	1.8	2.9	0.25	1,133	55.8a	136	74.3
ANOVA										
Nutrient solution (NS)		**	ns	ns	ns	ns	***	ns	ns	ns
Rootstock (R)		***	***	***	***	ns	*	***	***	*
R × NS		ns	ns	ns	ns	ns	ns	*	ns	ns

N, P, K, Ca, Mg, and Na in g 100 g^−1^ DW; Fe, Cu, Mn, Zn, and B in mg kg^−1^ DW. ^*^p < 0.05; ^**^p < 0.01; ^***^p < 0.001. ns, not significant. Different letters indicate significant differences according to Duncan’s multiple range test at the 95% CI.

**Figure 5:**
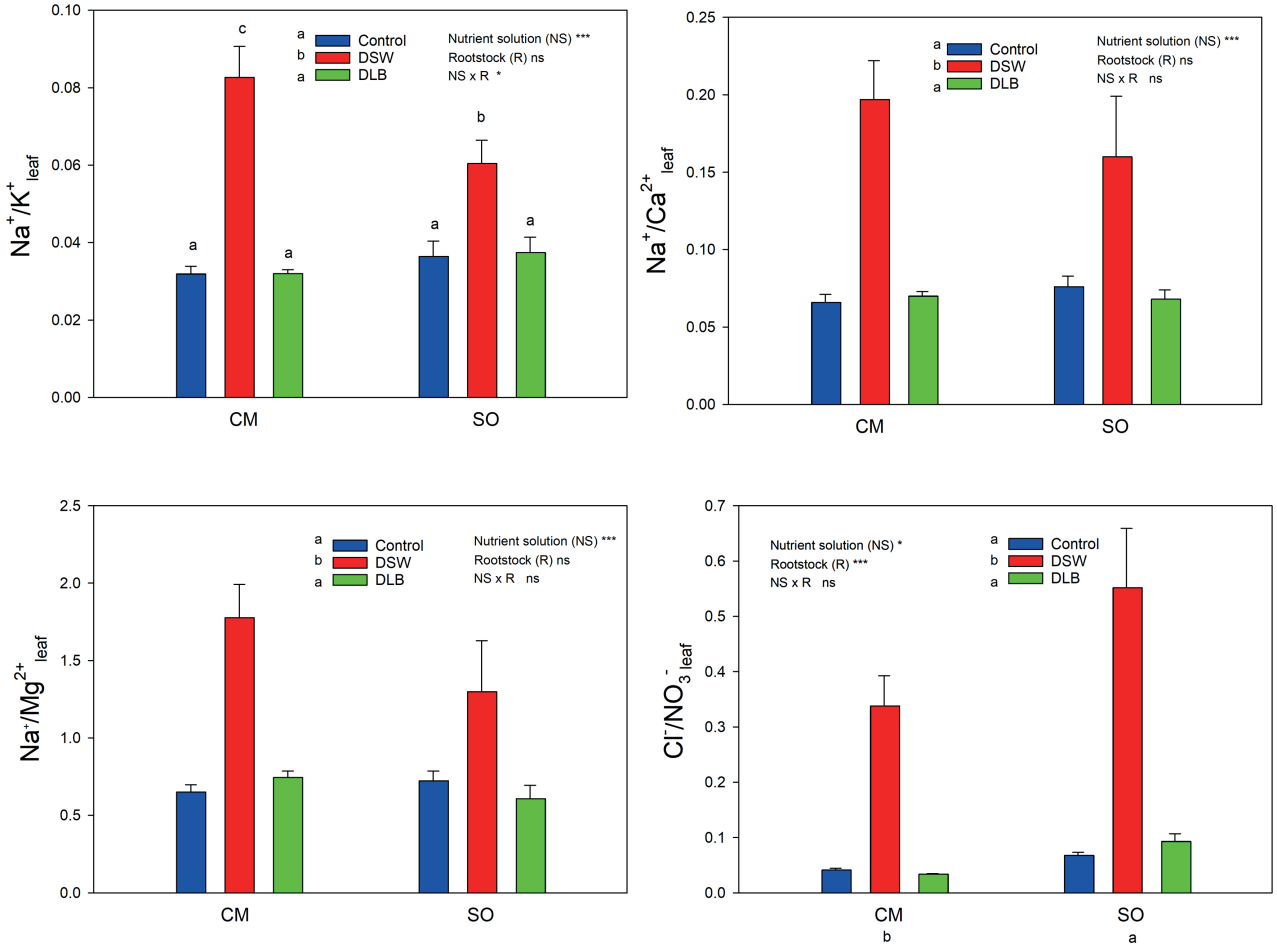
Effects of 7 months of irrigation with nutrient solutions of different characteristics—Control, DSW, and DLB—on different ratios of ionic concentrations (Na^+^ or Cl^−^ and essential nutrients) in leaves of ‘Verna’ lemon plants grown on two rootstocks, *Citrus macrophylla* (CM) and sour orange (SO). Different letters indicate significant differences according to Duncan’s multiple range test at the 95% confidence level.

**Figure 6:**
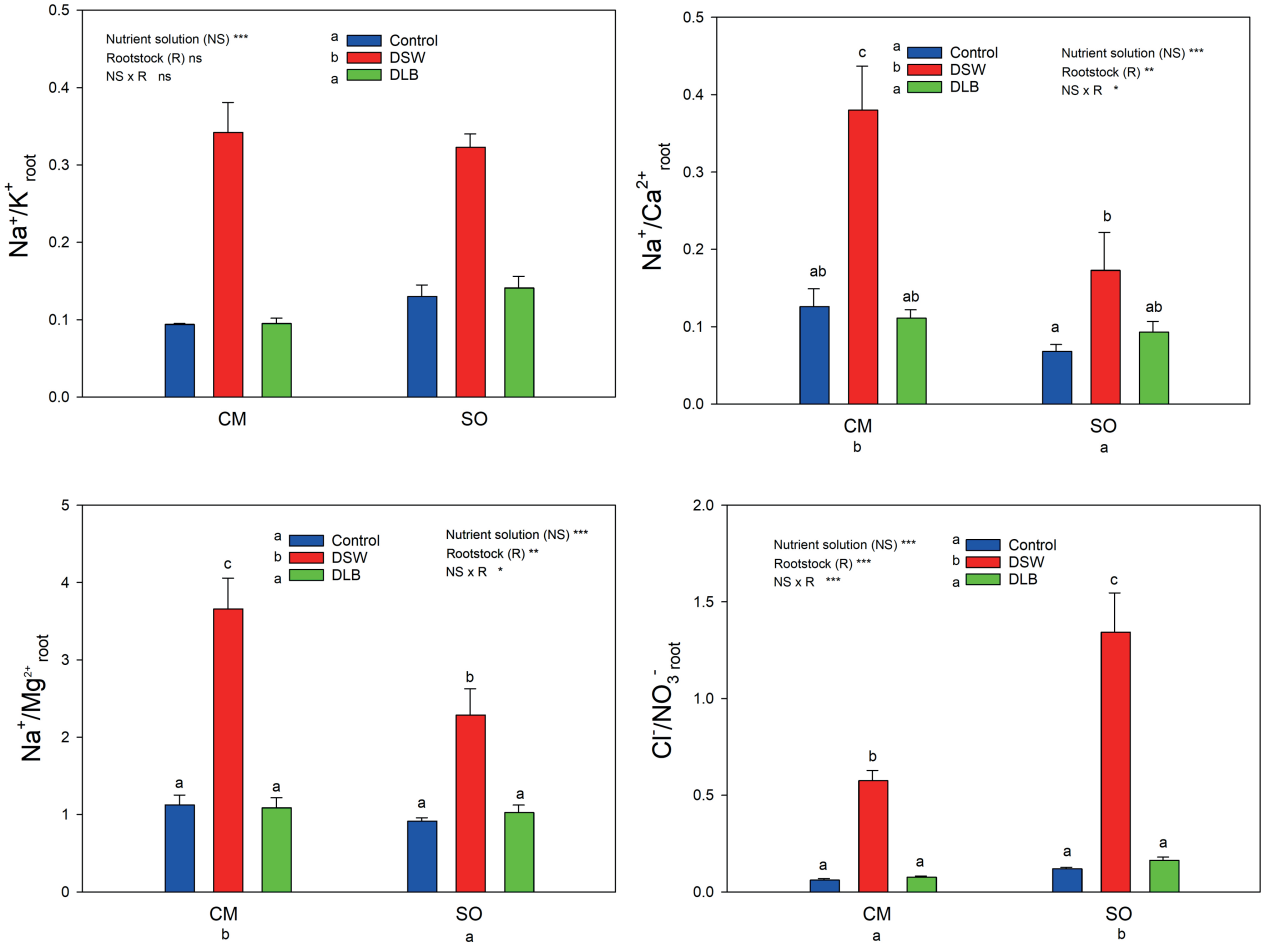
Effects of 7 months of irrigation with nutrient solutions of different characteristics—Control, DSW, and DLB—on different ratios of ionic concentrations (Na^+^ or Cl^−^ and essential nutrients) in roots of ‘Verna’ lemon plants grown on two rootstocks, *Citrus macrophylla* (CM) and sour orange (SO). Different letters indicate significant differences according to Duncan’s multiple range test at the 95% confidence level.

The 7 months of irrigation with nutrient solutions of different mineral composition especially affected the nitrogen concentrations in the plants, DSW-irrigated plants having the lowest N concentrations in leaves and roots (Tables 4 and 5). Leaves of DSW-irrigated plants, on both CM and SO, had the highest Cl^−^/NO_3_^−^ ratios (Figure 5), SO being the rootstock that gave the highest ratios, due to low concentrations of NO_3_^−^ rather than high concentrations of Cl^−^. Roots of DSW-irrigated plants also had significantly higher Cl^−^/NO_3_^−^ ratios than those of Control or DLB-irrigated plants, with significantly higher ratios in SO plants than in CM plants irrigated with DSW (Figure 6).

Leaves and roots of DSW-irrigated plants had the highest Na^+^/K^+^ ratios (Figures 5, 6), mainly due to their high Na^+^ concentrations. Moreover, leaves of plants grafted on CM had higher Na^+^/K^+^ ratios than those of SO plants (Figure 5). The DSW treatment reduced the Ca concentration in leaves but not in roots (Tables 4 and 5); however, the Na^+^/Ca^2+^ ratios were significantly higher in both leaves and roots of DSW-irrigated plants than in those of Control plants (Figures 5, 6), being higher in roots of CM plants than in roots of SO plants. Similarly, the Na^+^/Mg^2+^ ratios were highest in DSW-irrigated plants due to the high Na^+^ concentrations (higher in CM roots than in SO roots).

### Physiological and Biochemical Determinations

Throughout the experiment, some plant physiological parameters were determined periodically, to check the behavior of the plants. In order to simplify this information, the mean values of the parameters recorded during the last stage of the experiment are presented in Table 6. The plant water status was not modified by the irrigation treatments but it did depend on the rootstock. Although they were well irrigated, SO plants had lower water potentials than CM plants, but not higher osmotic potentials—so these plants had lower leaf turgor potential values (Table 6).

**Table 6:** Effects of irrigation with nutrient solutions of different characteristics—Control, DSW, and DLB—and the rootstock on the plant water relations, gas interchange, and chlorophyll fluorescence parameters of ‘Verna’ lemon plants grown on two rootstocks, *Citrus macrophylla* (CM) and sour orange (SO).

Nutrient solutions (NS)		Ψ_leaf_	Ψ_Π_	Ψ_P_	*A*	*g_s_*	*E*	*A*/*g_s_*	*A*/*E*	Φ_PSII_	F_v_’/F_m_′	qP	A/Φ_PSII_
Control		−0.81	−1.93	1.14	1.89b	0.011	0.45	211b	3.51b	0.38	0.59	0.68	4.83b
DSW		−0.80	−1.91	1.10	0.89a	0.012	0.27	108a	2.00a	0.41	0.63	0.67	2.18a
DLB		−0.76	−1.82	1.02	1.79b	0.011	0.38	152a	4.33b	0.40	0.60	0.69	4.62b
Rootstock (R)													
*Citrus macrophylla* (CM)		−0.73	−1.91	1.18	1.80	0.013	0.40	153	2.70	0.40	0.58	0.68	4.62
Sour orange (SO)		−0.85	−1.86	1.00	1.25	0.009	0.33	161	3.86	0.40	0.63	0.67	3.14
R × NS													
CM	Control	−0.75	−2.01	1.26	1.93	0.011	0.45	206	2.78ab	0.39	0.57	0.69	4.88bc
	DSW	−0.75	−1.87	1.12	1.40	0.013	0.27	125	2.47ab	0.41	0.62	0.67	3.44b
	DLB	−0.68	−1.85	1.16	2.07	0.015	0.49	128	2.85abc	0.39	0.56	0.70	5.54c
SO	Control	−0.87	−1.85	1.02	1.85	0.010	0.45	216	4.23bc	0.38	0.61	0.67	4.79bc
	DSW	−0.85	−1.94	1.09	0.39	0.010	0.28	92	1.53a	0.41	0.63	0.68	0.93a
	DLB	−0.83	−1.79	0.88	1.52	0.008	0.26	175	5.81c	0.41	0.64	0.68	3.70b
ANOVA													
Nutrient solution (NS)		ns	ns	ns	***	ns	ns	**	*	ns	ns	ns	*
Rootstock (R)		**	ns	**	*	*	ns	ns	ns	ns	*	ns	*
R × IW		ns	ns	ns	ns	ns	ns	ns	*	ns	ns	ns	*

The data are the means of all determinations made during the last 40 days of the experiment. Ψ_leaf_: leaf water potential (MPa), Ψ_Π_: leaf osmotic potential (MPa), Ψ_P_: leaf turgor potential (MPa), *A*: photosynthetic rate (μmol CO_2_ m^−2^ s ^−1^), *g_s_*: stomatal conductance of H_2_O (mol H_2_O m^−2^ s ^−1^), *E*: transpiration rate (mmol H_2_O m^−2^ s ^−1^), *A*/*g_s_*: intrinsic water use efficiency (μmol CO_2_ mol H_2_O^−1^), *A*/*E*: instantaneous water use efficiency (mmol CO_2_ mol H_2_O^−1^), Φ_PSII_: photochemical efficiency of PSII, F_v_’/F_m_′: efficiency of the PSII antennas, qP: photochemical quenching. ^*^p < 0.05; ^**^p < 0.01; ^***^p < 0.001. ns, not significant. Different letters indicate significant differences according to Duncan’s multiple range test at the 95% CI.

Regardless of the irrigation treatment, trees grafted on CM had the greatest photosynthetic rate (*A*) and stomatal conductance (*g_s_*), although no significant differences between the rootstocks were found for the rest of the gaseous interchange parameters (Table 6). The irrigation with DSW decreased *A*, but did not modify *g_s_* or transpiration rate (*E*). The intrinsic water use efficiency (*A*/*g_s_*) and the instantaneous water use efficiency (*A*/*E*) were decreased by DSW, while the DLB treatment decreased *A*/*g_s_*. On the other hand, no changes due to the irrigation with DSW or DLB were observed for the chlorophyll fluorescence parameters studied (Table 6). However, due to the reduction in the photosynthetic rate with the DSW treatment, the A/Φ_PSII_ ratio also decreased in DSW-irrigated plants, especially in those grafted on SO.

‘Verna’ lemon plants grafted on SO had lower chlorophyll concentrations in their leaves than CM-grafted plants, but no effect of the irrigation treatments on the leaf chlorophyll level was observed (Table 7). The proline concentration was decreased with respect to the Control in DSW-irrigated plants grafted on CM, but similar values were found for the rest of the treatments. However, QAC were significantly more abundant in plants grafted on SO and irrigated with the DSW (Table 7). These plants also had a higher MDA concentration in the leaves than plants grafted on SO and irrigated with the Control or DLB treatment. Regardless of the rootstock, a general increase in H_2_O_2_ was also detected in DSW-irrigated plants, relative to Control plants (Table 7).

**Table 7:** Effects of 7 months of irrigation with nutrient solutions of different characteristics—Control, DSW, and DLB—and the rootstock on H_2_O_2_, malondialdehyde (MDA), proline, and quaternary ammonium compounds (QAC) accumulation and the total chlorophyll in leaves of ‘Verna’ lemon plants grown on two rootstocks, *Citrus macrophylla* (CM) and sour orange (SO).

Nutrient solution (NS)		Total chlorophyll	Proline	QCA	H_2_O_2_	MDA
Control		10.1	8.1	3.45	89.8a	24.4a
DSW		10.0	7.2	3.66	135.7b	27.2b
DLB		10.4	7.3	3.24	94.4ab	24.7ab
Rootstock (R)						
*Citrus macrophylla* (CM)		10.9	7.8	3.41	91.9	25.4
Sour orange (SO)		9.4	7.2	3.50	121.3	25.4
R × NS						
CM	Control	10.5	8.7b	3.62ab	90.7	26.6bc
	DSW	11.0	6.4a	3.19a	112.2	24.6ab
	DLB	11.3	8.3ab	3.42a	72.8	25.1b
SO	Control	9.8	7.4ab	3.28a	88.9	22.3a
	DSW	9.1	8.0ab	4.14b	159.2	29.7c
	DLB	9.4	6.3a	3.06a	115.9	24.2ab
ANOVA						
Nutrient solution (NS)		ns	ns	ns	*	*
Rootstock (R)		***	ns	ns	ns	ns
R × NS		ns	*	**	ns	**

H_2_O_2_ (μg g^−1^ FW), MDA (μmol kg^−1^ FW), proline (mg g^−1^ FW), QAC (mg g^−1^ FW), and chlorophyll (mg g^−1^ DW). ^*^p < 0.05; ^**^p < 0.01; ^***^p < 0.001. ns, not significant. Different letters indicate significant differences according to Duncan’s multiple range test at the 95% CI.

## Discussion

The effect of irrigation with DSW or DLB on the plants was the result of the combined effects of high concentrations of Cl^−^, Na^+^, and B (DSW), or a moderate concentration of B (DLB; Table 1). After 7 months of exposure to DSW, the total dry weight of ‘Verna’ lemon trees (in particular, those grafted on CM) was lower than that of the Control trees, although this decrease was not significant (Table 3). The irrigation with DSW inhibited shoot growth (lower leaf size and lateral stems weight) rather than root growth in CM plants. The plant growth reduction caused by DSW could have been due to the high levels of Na^+^, Cl^−^, and B accumulated in the leaves (Figures 1, 2), produced by the high concentrations of these elements in the nutrient solution (Table 1). Citrus plants are B and salt-sensitive and high concentrations of Cl^−^, Na^+^, and B in the irrigation water reduce plant growth due to their accumulation in leaves (Maas, 1993; Papadakis et al., 2003; Al-Yassin, 2004; García-Sánchez et al., 2005; Syvertsen and Garcia-Sanchez, 2014).

The concentration of Cl^−^ in DSW was 300 mg L^−1^ (Table 1), above the thresholds proposed by Levy and Syvertsen (2004) (152 mg L^−1^) and Ayers and Westcot (1985) (238 mg L^−1^) to produce injury in citrus. The concentration of Na^+^ in DSW (167 mg L^−1^, Table 1) exceeded by 45% the toxic threshold (115 mg L^−1^) proposed for the most sensitive varieties of citrus (Grattan et al., 2015). The toxicity risk due to the concentrations of Cl^−^ and Na^+^ in the DSW was considered as moderate (Ayers and Westcot, 1985). Compared to the high concentrations of Na^+^ and Cl^−^, the B concentrations were much lower (Table 1). However, these low concentrations can negatively affect plant growth. The B concentration in the Control treatment was 0.27 mg L^−1^, below the threshold of 0.50 mg L^−1^ proposed for citrus (Voutchkov and Semiat, 2008; Martínez-Alvarez et al., 2017), in the DLB treatment it was 0.50 mg L^−1^, and in the DSW treatment, 1.23 mg L^−1^, exceeding the threshold proposed by these authors.

With these concentrations of phytotoxic elements in the nutrient solutions, after 7 months of irrigation, DSW-irrigated substrates (Table 2) and, as a consequence, DSW-treated plants, had the highest Cl^−^, Na^+^ and B concentrations, with significant differences in their accumulation in the plant tissues between CM and SO plants (Figures 1, 2). In spite of the high concentration of Cl^−^ in DSW, after 7 months of irrigation, the leaf toxic threshold for Cl^−^ of 6 mg g^−1^ DW proposed by Romero-Trigueros et al. (2014) was exceeded only (and slightly) in the CM plants, and the threshold of 10 mg g^−1^ DW proposed by Grattan et al. (2015) was not reached. Besides, after 7 months, SO plants had accumulated significantly more Cl^−^ than CM plants in roots, but less in leaves (Figure 2), avoiding damage to their photosynthetic apparatus. Citrus salt tolerance is related to the ability to restrict the uptake of saline ions and/or their transport from roots to shoots (Cerdá et al., 1979; Walker et al., 1983; Zekri and Parsons, 1992). So, based on the Cl^−^ partitioning in the plant, ‘Verna’ lemon plants grafted on SO were more tolerant, since Cl^−^ was more effectively retained in their roots, with less translocation of Cl^−^ to the shoot and lower amounts reaching the leaves than in CM plants. The high Na^+^ level in DSW produced, for both the CM and SO rootstocks, an increase in the leaf concentration to above 2.5 mg g^−1^ DW, the Na^+^ threshold for leaf phytotoxicity proposed for citrus (Grattan et al., 2015). Moreover, plants grafted on CM accumulated in their leaves and, particularly, in their roots more Na^+^ than those grafted on SO (Figure 2). In citrus, Cl^−^, rather than Na^+^, has been related to leaf injury and reductions of growth (Grieve and Walker, 1983; Walker et al., 1983; Maas, 1993); so, the salt tolerance of citrus species is sometimes determined by their capacity for Cl^−^ exclusion. However, other authors have associated the adverse effects of high external Na^+^ and Cl^−^ concentrations on citrus with the foliar accumulation of Na^+^ (Behboudian et al., 1986; Lloyd et al., 1987). Moreover, it has been widely described that the ability of citrus plants to tolerate excessive amounts of Cl^−^ and Na^+^ varies widely among rootstocks, since they differ in their ion accumulation or exclusion, as has been shown in this experiment (Figure 2). While CM is considered an accumulator of both Cl^−^ and Na^+^, SO is considered as a good Cl^−^ and Na^+^ excluder (Nieves et al., 1991; Storey and Walker, 1999). In our experiment, the leaf Na^+^ concentrations reached in both CM and SO plants exceeded 2.5 mg g^−1^ DW, the threshold of phytotoxicity proposed by Grattan et al. (2015), while the Cl^−^ concentrations in leaves were higher than the threshold of Romero-Trigueros et al. (2014) only in CM plants irrigated with DSW. So, both Cl^−^ and Na^+^ were high enough to produce foliar injuries in CM plants, but only the high foliar Na^+^ level seems to have been responsible for foliar damage in SO plants. Besides, unlike what happened in ‘Verna’ lemon plants grafted on CM, the growth of plants grafted on SO was not reduced by DSW. Hence, the decrease in growth of ‘Verna’ lemon plants irrigated with DSW was probably related more to the foliar concentration of Cl^−^ than to the foliar Na^+^ concentration.

The different nutrient solutions produced different B accumulation in the plants: the leaves of plants receiving the Control treatment had 82 mg B kg^−1^ DW, below the leaf B threshold of 100 mg kg^−1^ DW, above which damage can occur (Embleton et al., 1973). The leaves of plants irrigated with DLB had significantly increased B concentrations: 125 and 184 mg kg^−1^ DW in plants grafted on CM and SO, respectively (Figure 1). These B concentrations are in the range of 100–300 mg kg^−1^ DW that, according to Grattan et al. (2015), could produce slight to moderate injuries. Plants irrigated with DSW had significantly more B in their leaves than Control or DLB-irrigated plants: 209 and 303 mg kg^−1^ DW in CM and SO plants, respectively. These concentrations are in the upper part of the range proposed by Grattan et al. (2015), but with clear differences between the rootstocks. In fact, the B levels in leaves of CM plants were well below the 250–260 mg kg^−1^ DW range for which Embleton et al. (1973) proposed that toxicity occurs in citrus, whereas the B levels in leaves of SO plants were well above this range. These results indicate that the leaf B concentration of lemon plants treated with excess B was rootstock-dependent. In general, with DLB and DSW irrigation, leaves of SO plants accumulated significantly more B than leaves of CM plants. The differing responses of the rootstocks to different external B concentrations could be due to the involvement of distinct mechanisms in the uptake and transport of B, which may include molecular factors as well as physiological ones. Boron uptake by roots can take place by passive diffusion of boric acid, facilitated diffusion of boric acid *via* channels, or uptake of the borate anion *via* passive and/or active transporters (Yoshinari and Takano, 2017). The B uptake capacity of the B transport channels in the root (*NIP5* and *PIP1* genes) is blocked in CM plants under high B fertilization (Martinez-Cuenca et al., 2015). This could have contributed to the lower concentration of B found in roots of CM plants irrigated with DSW, relative to SO plants. Once B enters the roots, it is transported by the transpiration stream toward the upper part of the tree (Papadakis et al., 2004), so a lower transpiration rate decreases the entry of B into the plants (Mesquita et al., 2016). The lower B concentration in leaves of CM plants could also be related to the transpiration rate; however, the transpiration rate did not differ significantly between the rootstocks during the experiment (Table 6). The greater accumulation of B in SO plants could have been due, among other factors, to the fact that the root/leaf ratio was higher in SO plants than in CM plants (0.60 and 0.41, respectively, Table 3). Hence, for the same B uptake capacity (per gram of root), a greater amount of roots and/or a lower leaf biomass could have produced a greater concentration effect for B in the leaves of SO plants.

With regard to the effect of B on plant growth, species and genotypes sensitive to excess B generally have higher concentrations in leaves and shoots than tolerant genotypes (Nable et al., 1997; Camacho-Cristóbal et al., 2008; Simón-Grao et al., 2018). However, our results for ‘Verna’ lemon plants grafted on CM and SO rootstocks show that plants on the rootstock that was less tolerant of the irrigation with DSW (CM) had lower concentrations of B in their leaves; so, the shoot growth reduction due to irrigation with DSW was not directly related to the concentration of B in the leaves. Whereas a B concentration of 209 mg kg^−1^ in the leaves of plants grafted on CM significantly reduced the shoot biomass, by 19%, a higher concentration in the leaves of plants grafted on SO (303 mg kg^−1^) did not. It has also been found in citrus plants that similar concentrations of B in leaves produce different growth responses depending on the rootstock. So the foliar concentration is not the only factor that determines the relative B tolerance of different rootstocks (Gimeno et al., 2012; Mesquita et al., 2016) and different rootstock-dependent mechanisms can mitigate the negative effects of high foliar B levels (Gimeno et al., 2012).

The irrigation with DSW or DLB also produced visual damage to leaves. Plants irrigated with DSW showed a high percentage of leaves with visual damage, with burns at the apex and edges of leaves (Figures 3, 4). The percentage of injured leaves was lower in DLB-irrigated plants, but still significantly greater than in Control plants. It could be thought that these burns were due to B toxicity. However, it has been reported that the visual symptoms of high B in citrus leaves start as a yellowing of the apex and edges of mature leaves and later spread to the rest of the leaf, in the form of chlorotic and/or necrotic patches (Gimeno et al., 2012; García-Sánchez et al., 2020). The damaged leaves in this experiment were completely green, without any chlorotic patches, so the burns do not appear to have been due to excess B. Rather, they may have been due to excess Cl^−^ or Na^+^ or a combination of all three ions, since DLB-irrigated plants also had a high percentage of injured leaves and did accumulate B but not Cl^−^ or Na^+^ (Figures 1, 2). With DSW irrigation, both Cl^−^ and Na^+^ were high enough to produce foliar injuries in CM plants, but only the high foliar Na^+^ level seems to have been responsible for the foliar damage in SO plants.

The citrus response to the levels of Cl^−^, Na^+^, and B in DSW was rootstock-dependent and different physiological, biochemical and nutritional alterations could explain the differing behaviors of CM and SO-grafted plants. Irrigation with DSW significantly reduced the photosynthetic rate (Table 6) but the stomatal conductance was not responsible for this because there was an increase in Ci (data not shown) in CM and SO plants irrigated with DSW. Other internal, non-stomatal factors could have contributed to the decline in photosynthesis. In several studies of citrus plants, it was established that non-stomatal factors could have a marked influence on the decline in photosynthesis caused by Cl^−^, Na^+^, or B toxicity (Keles et al., 2004; Papadakis et al., 2004; Garcia-Sanchez and Syvertsen, 2006; Pérez-Pérez et al., 2007). Some effects of salinity or B toxicity—such as alterations of the carboxylation efficiency, the photochemical efficiency of photosystem II, or the chlorophyll concentration, oxidative damage, reduced activities of photosynthetic enzymes, an impaired electron transport capacity, and alterations to the leaf structure and chloroplast ultrastructure (Keles et al., 2004; Papadakis et al., 2004; López-Climent et al., 2008; Han et al., 2009)—could be partly responsible for the lower rate of photosynthesis. In our experiment, no reduction of the chlorophyll concentration by the irrigation treatments was detected and the photochemical machinery was not damaged by the high B_,_ Cl^−^, or Na^+^ concentrations in leaves of DSW-irrigated plants (as Φ_PSII_, the proportion of the absorbed energy being used in photochemistry, was not modified; Table 3). Under salinity stress or with a high B concentration, the photosynthetic metabolism can be altered and this may lead to overproduction of ROS and H_2_O_2_ (Arbona et al., 2003). The ROS produced under stress react with many different molecules, yielding several oxidative by-products and a sub-product, MDA, widely used as a marker for oxidative damage (Arbona et al., 2003; Hossain et al., 2009). Due to the reduction of the photosynthetic rate, the A/Φ_PSII_ ratio decreased in DSW-irrigated ‘Verna’ lemon plants, especially those grafted on SO. When the A/Φ_PSII_ ratio decreases, the generation of ROS increases since O_2_ operates as an alternative acceptor, depleting the electron excess that is not utilized in metabolic processes (Cakmak and Römheld, 1997). This greater oxidative stress in plants grafted on SO and irrigated with DSW was manifested as the increased concentrations of H_2_O_2_ and MDA in leaves (Table 7). The greater decrease in the A/Φ_PSII_ ratio and the increase in leaf MDA concentrations in SO plants irrigated with DSW suggest that ‘Verna’ lemon plants grafted on the SO rootstock have an inefficient antioxidant system and were not able to cope with the ROS produced as a consequence of the irrigation with DSW. In other work, Simón-Grao et al. (2018) suggested that ungrafted plants of SO exposed to a B concentration (10 mg L^−1^) higher than the ones we used had an efficient antioxidant system that was able to deal with the ROS, ultimately detoxifying the B. These different responses could be attributed to differences in the experimental conditions—B concentration, plant age, grafted/ungrafted plants, environmental conditions (normal/high temperature), etc.—but also to the special characteristics of the nutrient solutions used in our experiment, since they were desalinated seawaters, with high B levels but also high concentrations of Cl^−^ and Na^+^. The higher QAC levels detected in SO plants under the DSW treatment (Table 7) probably had an antioxidant role, in view of the greater oxidative damage that these plants suffered. Simón-Grao et al. (2018) found that QAC were the only organic solutes involved in the adaptation of citrus to B toxicity, and their role is related to the protection of thylakoid membranes (Genard et al., 1991). This could help to explain why SO plants maintained their photochemical efficiency (Φ_PSII_) at levels similar to that of Control plants despite having the highest B concentration in their leaves.

An imbalance of essential nutrients could also have been involved in the response of the plants to irrigation with DSW and DLB. Grattan and Grieve (1992) established that, when the soil solution has high Na^+^ and Cl^−^ concentrations, nutrient uptake by plants can be reduced, either by direct competition between ions or by the decreased osmotic potential of the solution, which reduces the mass flow of mineral nutrients to the root surface. In consequence, the nutritional balance of plants may be altered, with high Na^+^/Ca^2+^, Na^+^/K^+^, Na^+^/Mg^2+^, Cl^−^/NO_3_^−^, and Cl^−^/H_2_PO_4_^−^ ratios. Also, a high concentration of B in the nutrient solution can modify the P, K, and Ca nutrition (Kaya et al., 2011). In all treatments, the leaf Ca^2+^, K^+^, Mg^2+^, and P concentrations were within the optimum range and leaf N was above the optimum range for bearing citrus (Embleton et al., 1973). However, the irrigation with different nutrient solutions altered the mineral nutrition in roots and leaves, and these modifications depended not only on the nutrient solution but also on the rootstock (Tables 4 and 5). The irrigation with DSW significantly reduced the Ca^2+^ concentration in leaves of CM and SO plants, but not in roots, indicating that root-to-shoot Ca^2+^ translocation was inhibited in these plants. Calcium uptake and translocation from roots to leaves in citrus can be inhibited by salinity (Cámara-Zapata et al., 2004). The low Ca^2+^ concentrations in leaves of DSW-irrigated plants resulted in higher Na^+^/Ca^2+^ ratios, not only in leaves, for both rootstocks, but also in roots (mainly in CM plants), due to the high Na^+^ concentration in these organs. There is competition between Na^+^ and Ca^2+^ ions for anionic sites, Ca^2+^ being displaced by Na^+^ in the cell walls, producing the necrotic burns in leaves that are characteristic of Na^+^ toxicity (Zid and Grignon, 1985). The Na^+^/Ca^2+^ ratio is an important variable that is often overlooked when evaluating the effect of salinity on plants, since when it becomes too great it is a sign that excessive amounts of Na^+^ have been taken up by the roots and transported to the leaves (Maas, 1993). A high Na^+^ concentration in the medium can also result in plant K^+^ deficiency, although a decline in K^+^ does not always occur in citrus leaves under salinity (Behboudian et al., 1986). In this experiment, irrigation with DSW (167 mg L^−1^ Na^+^) did not reduce the K^+^ concentration in the roots or leaves of CM or SO plants (Tables 4 and 5); however, DSW-irrigated plants had a significantly increased Na^+^/K^+^ ratio in their roots and leaves due to the high Na^+^ concentrations in the plants. Although the K^+^ and Na^+^ concentrations were higher in CM than in SO roots, the Na^+^/K^+^ ratio was similar in the roots of the two rootstocks. Leaves of CM plants also had more K^+^ and Na^+^ than those of SO plants, but with the DSW treatment, CM plants had a higher Na^+^/K^+^ ratio than SO plants (Figure 5). So, the K^+^ translocation to the aerial part was more limited in CM than in SO plants. The high Cl^−^ concentration in the DSW treatment reduced the N concentration in leaves and roots (Tables 4 and 5), as has been observed previously in citrus under saline conditions due to antagonism between NO_3_^−^ and Cl^−^ ions at the transport sites in roots (Cerezo et al., 1997; Cámara-Zapata et al., 2004). In our study, leaves and roots of ‘Verna’ lemon plants grafted on SO and irrigated with DSW had the highest Cl^−^/NO_3_^−^ ratios (Figures 5, 6), SO plants showing higher uptake and transport of Cl^−^ than CM plants. As plants grafted on CM had higher concentrations of N in the leaves and roots than those grafted on SO, this could have helped to mitigate the adverse effects of higher concentrations of Cl^−^ in the leaves and roots.

## Conclusion

Desalinated seawater is an alternative source of irrigation water in some areas with serious water scarcity. In citrus, it should be used with caution since, due to its chemical composition, it can generate some phytotoxicity problems. Citrus plants are sensitive to Cl^−^, Na^+^, and B and these ions are present in DSW at levels high enough to produce toxicity in these plants. The plant responses to the irrigation treatments in this experiment were the result of the combined effects of high concentrations of Cl^−^, Na^+^, and B (DSW) or a moderate concentration of B (DLB). The behavior of DLB plants was similar to that of Control plants since the B that remains after the reduction process is low enough not to cause toxicity problems; so, the use of systems to reduce the high B concentration of DSW should be considered when using DSW for irrigation of B-sensitive crops.

The citrus response to the levels of Cl^−^, Na^+^, and B of DSW, was rootstock-dependent; hence, the plant behavior (involving nutritional, physiological, and biochemical alterations) differed between the CM and SO-grafted plants. The growth reduction of ‘Verna’ lemon plants irrigated with DSW and grafted on the CM rootstock was related to the foliar accumulation of Na^+^ and Cl^−^, which produced high Na^+^/K^+^ and Na^+^/Ca^2+^ ratios. The growth of plants grafted on SO was not affected by DSW (the Cl^−^ threshold of phytotoxicity was not reached); so, the decreased growth was related more to the Cl^−^ concentration than to that of Na^+^. Besides, the growth reduction was also not related to the B concentration since SO-grafted plants, with the highest concentrations of B, did not exhibit diminished growth. However, these high B concentrations produced high oxidative stress, which could have been partly countered by the increase in QAC levels; this may help to explain why SO plants maintained their high photochemical efficiency.

The difference in behavior of citrus plants grafted on different rootstocks when DSW is used for irrigation under high temperature conditions is of great interest since this information could be relevant to decide which plant material to use in citrus plantations in the future, when temperatures rise due to CC and farmers will be forced to use DSW for citrus irrigation.

## Data Availability Statement

The raw data supporting the conclusions of this article will be made available by the authors, without undue reservation.

## Author Contributions

JN and VA: conceptualization, writing—original draft preparation, and writing—review. JN, VA, JR, and PB: methodology. JN, VA, and JR: formal analysis and data interpretation. JN, PB, and VA: investigation. JN and PB: editing. JN: funding acquisition. All authors contributed to the article and approved the submitted version.

## Funding

This study was supported by the Ministry for Science and Innovation (MCIN, Spain), the State Research Agency (AEI, Spain), the European Regional Development Fund (ERDF, EU) under the project SEARRISOST (RTC-2017-6192-2), and the European Social Fund and the ERDF, EU, under the project FEDER1420-24.

## Conflict of Interest

The authors declare that the research was conducted in the absence of any commercial or financial relationships that could be construed as a potential conflict of interest.

## Publisher’s Note

All claims expressed in this article are solely those of the authors and do not necessarily represent those of their affiliated organizations, or those of the publisher, the editors and the reviewers. Any product that may be evaluated in this article, or claim that may be made by its manufacturer, is not guaranteed or endorsed by the publisher.

## Footnotes

^1^ http://www.ipcc.ch/
